# The incidence and mortality of connective tissue diseases: a population-level cohort study in England from 2012 to 2023

**DOI:** 10.1093/rheumatology/keaf414

**Published:** 2025-08-04

**Authors:** Samir Patel, Mark D Russell, Katie Bechman, Maryam A Adas, Zijing Yang, Edward Alveyn, Chris Wincup, Alex Dregan, Kate Bramham, Sam Norton, James B Galloway, Patrick Anthony Gordon

**Affiliations:** Centre for Rheumatic Diseases, King’s College London, London, UK; Medical Subspecialties Institute, Cleveland Clinic London, London, UK; Centre for Rheumatic Diseases, King’s College London, London, UK; Centre for Rheumatic Diseases, King’s College London, London, UK; Centre for Rheumatic Diseases, King’s College London, London, UK; Centre for Rheumatic Diseases, King’s College London, London, UK; Centre for Rheumatic Diseases, King’s College London, London, UK; Centre for Rheumatic Diseases, King’s College London, London, UK; Centre for Rheumatic Diseases, King’s College London, London, UK; Centre for Rheumatic Diseases, King’s College London, London, UK; Medical Subspecialties Institute, Cleveland Clinic London, London, UK; Centre for Rheumatic Diseases, King’s College London, London, UK; Centre for Rheumatic Diseases, King’s College London, London, UK; Centre for Rheumatic Diseases, King’s College London, London, UK

**Keywords:** connective tissue diseases, systemic lupus erythematosus, Sjögren’s syndrome, systemic sclerosis, idiopathic inflammatory myopathies, mixed connective tissue disease, incidence, mortality

## Abstract

**Objectives:**

The reported incidence and mortality of connective tissue diseases (CTDs) in England has been inconsistent in the literature. Our objective was to describe current trends in the incidence and mortality of systemic lupus erythematous (SLE), Sjögren’s disease (SjD), systemic sclerosis (SSc), idiopathic inflammatory myopathies (IIM) and mixed connective tissue disease (MCTD).

**Methods:**

We conducted a retrospective population-level study using primary care records in England via the Clinical Practice Research Datalink. We included individuals ≥18 years old with new CTD diagnoses between 2012 and 2023. Our outcomes were incidence and all-cause mortality, which included age-standardized mortality rates (ASMRs), standardized mortality ratios and hazards over time using flexible parametric models.

**Results:**

There was a total of 22 829 incident CTD diagnoses (81.1% female, median age 57). The age and sex-standardized incidence of SLE and SSc fell over the study period 2012–2023 (SLE: 4.32–3.29 per 100 000 person years [py] and SSc: 2.33–1.86 per 100 000 py), whereas SjD and MCTD incidence remained relatively stable. In contrast, IIM diagnoses rose from 3.23 to 4.31 per 100 000 py. ASMRs across the study period were highest for IIM (27.83 per 1000 py), followed by SSc (24.43), SLE (16.74), MCTD (16.27) and lowest for SjD (9.70).

**Conclusion:**

Our findings indicated a fall in the incidence of SLE, a simultaneous rise in IIM incidence, and high all-cause mortality within IIM and SSc cohorts. Our study acknowledges the changing landscape of CTDs in England and will aid healthcare resource planning for this vulnerable population.

Rheumatology key messages

- From 2012 to 2023, the age and sex-standardized incidence of SLE fell and IIM rose.
- IIM and SSc cohorts had the highest mortality (ASMR, SMR and risk of death early in disease course).

## Introduction

High quality, contemporary epidemiology frames medical paradigms for both clinical and economic purposes. In general, there is a scarcity of comparative analyses that span multiple connective tissue diseases (CTDs) in the literature.

Systemic lupus erythematosus (SLE), Sjögren’s disease (SjD), systemic sclerosis (SSc), idiopathic inflammatory myopathies (IIM) and mixed connective tissue disease (MCTD) are rare CTDs affecting ∼5–97 per 100 000 people. Over the last decade, updated international classification criteria have better defined these conditions based on growing bodies of evidence [1–4]. Increased awareness among clinicians and advances in diagnostic technologies such as autoantibody testing and PET have also likely contributed to earlier recognition of these conditions and timelier referral into Rheumatology care pathways. Furthermore, the repertoire of therapeutic options for CTDs has grown in recent years with the emergence and increased accessibility of biologic and targeted synthetic therapies.

The incidence of SLE, SjD, SSc, IIM or MCTD in England has not been described beyond 2019 [5, 6], nor has the effect of the COVID-19 pandemic on diagnoses or mortality. Additionally, the impact of biologic and synthetic therapies on survival remains unexplored in England, as mortality studies of CTDs are either scarce or pre-date these therapies [7–9]. Our objective was to explore trends in incidence and mortality across a broad range of CTDs, including SLE, SjD, SSc, IIM and MCTD, using a large longitudinal cohort in England from 2012 to 2023.

## Methods

### Data source

We implemented a retrospective population-level observational study using the Clinical Practice Research Datalink (CPRD), one of the world’s largest primary care electronic health record datasets. We used the CPRD Aurum dataset that holds anonymized data for ∼18 million currently contributing patients in the UK (24% of the population) from primary care practices that use EMIS Web^®^ software. It is broadly representative of the population in terms of age, sex and ethnicity and has been utilized for epidemiological studies across a wide array of conditions, including connective tissue diseases [5].

### Study population

Individuals were deemed eligible for inclusion if they were at least 18 years of age and had a new CTD diagnosis between 1 January 2012 to 31 December 2023. A minimum of 12 months prior registration with a CPRD-registered practice was required to reduce the risk of misclassifying prevalent cases as incident. We identified patients with incident SLE, SjD, SSc, IIM or MCTD based on the recording of a SNOMED code in the primary care records (code lists in Supplementary Table S1) for individuals without previously recorded diagnostic codes for that condition. Individuals could contribute to more than one CTD cohort if they had additional CTD diagnoses within the study period. We used similar methods of case ascertainment to previous studies and believed this approach reflected true CTD diagnoses as primary care physicians are unlikely to assign a CTD SNOMED code without Rheumatology or specialist review [5, 10]. Notably, our study did not include rheumatoid arthritis as it is a distinct autoimmune condition with a different disease trajectory and clinical management compared with SLE, SjD, SSc, IIM and MCTD.

### Variables and outcomes

Baseline characteristics were extracted for all participants, including age, sex (male/female) and ethnicity. Ethnicity was self-reported and defined according to major ethnic groups: Asian, Black, Mixed, Other and White. The appearance of a new diagnostic code was considered the index date of diagnosis. Dates of deaths were extracted from CPRD. Data for age, sex and mortality were complete and did not require imputation. Data on ethnicity, relevant medications and investigations (e.g. blood tests or imaging) were collected where available but had high levels of missingness so were not included in the main analyses. This was likely due to incomplete reporting or poor capture of data from secondary care.

### Statistical analysis

Baseline characteristics at diagnosis were presented for each condition. Temporal trends in incidence rates were described by year of diagnosis. We calculated incidence rates using incident CTD cases as the numerator and person-years from the CPRD population as the denominator for that calendar year. To describe summary trends in incidence over the study period, we calculated incidence rate ratios (IRRs) by dividing the most recent incident rate by the earliest. This approach allowed clearer interpretation of changes in incidence across distinct calendar years, especially given the varying temporal patterns by disease. Sensitivity analyses were also performed, restricting incident cases to individuals meeting at least one of the following criteria: (i) two CTD SNOMED codes recorded at different timepoints; (ii) one CTD SNOMED code and a DMARD prescription in primary care, or (iii) one CTD SNOMED code and three or more corticosteroid prescriptions in primary care.

Mortality rates for the study period were calculated by dividing the total number of deaths by total number of person-years at risk for each individual CTD cohort. Standardized rates for incidence and mortality were determined by applying direct age and sex standardization to the 2013 European Standard Population (ESP) [11]. Standardized mortality ratios (SMRs) were calculated by dividing the number of observed deaths by the number of expected deaths, derived from the Office of National Statistics (ONS) mortality data for the general population of England [12]. Counts of fewer than eight were redacted to ensure statistical disclosure control.

To explore how mortality risk changes over time from CTD onset, we estimated condition-specific hazard functions using restricted cubic splines within a flexible parametric survival model (Royston–Parmar). Follow-up began at the date of CTD diagnosis. CTD subtype (SLE, SjD, SSc, IIM, MCTD) was modelled to allow hazard ratios to vary over time since diagnosis, enabling estimation of time-varying effects for each condition on mortality risk. The number of degrees of freedom was selected based on best-fit models using the Bayesian information criterion. Models were adjusted for age at diagnosis and sex. Additionally, we conducted sensitivity analyses to explore the effect of co-occurring CTD diagnoses on mortality outcomes by excluding individuals with more than one CTD diagnosis. To assess the impact of the COVID-19 pandemic on mortality, we also reported mortality rates and SMRs for the period before and after the pandemic onset (2012–2019 and 2020–2023). Statistical analyses were performed using Stata 18 (StataCorp LLC, College Station, TX, USA).

### Patient and public involvement

Patients and the public were not involved in the design, conduct or reporting of this research, but are involved in the dissemination of its findings.

### Ethical approval

Patient consent was not required for this study as it used anonymized data from the CPRD. The CPRD Group has ethical approval from a National Research Ethics Service Committee for all observational research using anonymized CPRD data. Scientific approval was given by the CPRD Independent Scientific Advisory Committee for this study (protocol 23_003200), and all analyses were conducted in accordance with relevant data protection policies.

## Results

There was a total of 22 829 incident CTD diagnoses between 1 January 2012 and 31 December 2023. This encompassed 4937 incident SLE diagnoses, 9535 SjD diagnoses, 2946 SSc diagnoses, 4496 IIM diagnoses and 915 MCTD diagnoses. Baseline characteristics are summarized in Table 1. Median age at diagnosis was lowest for SLE (47; IQR 36–60), followed by MCTD (49; IQR 39–61), SjD (59; IQR 48–69), IIM (60; IQR 47–71), and SSc (60; IQR 49–71). IIM incidence had a similar split between sexes (54% female), whilst SLE, SjD, SSc and MCTD all had a female predominance (87.9%, 89.7%, 82.3% and 83.9%, respectively).

**Table 1.** Baseline characteristics of patients with incident CTDs in CPRD from 2012 to 2023; demographic data taken at time of diagnosis

	SLE	SjD	SSc	IIM	MCTD
	(*n* = 4937)	(*n* = 9535)	(*n* = 2946)	(*n* = 4496)	(*n* = 915)
Age at diagnosis, median (IQR), years	47 (36–60)	59 (48–69)	60 (49–71)	60 (47–71)	49 (39–61)
Age band at diagnosis, *n* (%)					
18–29	704 (14.3)	297 (3.1)	129 (4.4)	286 (6.4)	88 (9.6)
30–39	922 (18.7)	825 (8.7)	232 (7.9)	406 (9.0)	156 (17.0)
40–49	1103 (22.3)	1442 (15.1)	414 (14.1)	671 (14.9)	215 (23.5)
50–59	955 (19.3)	2324 (24.4)	634 (21.5)	876 (19.5)	185 (20.2)
60–69	666 (13.5)	2298 (24.1)	720 (24.4)	1001 (22.3)	158 (17.3)
70–69	431 (8.7)	1738 (18.2)	589 (20.0)	866 (19.3)	91 (9.9)
80+	156 (3.2)	611 (6.4)	228 (7.7)	390 (8.7)	22 (2.4)
Gender, *n* (%)					
Female	4338 (87.9)	8555 (89.7)	2425 (82.3)	2430 (54.0)	768 (83.9)
Ethnicity, *n* (%)					
White	2427 (49.2)	5533 (58.0)	1655 (56.2)	2425 (53.9)	447 (48.9)
Black	566 (11.5)	455 (4.8)	124 (4.2)	340 (7.6)	117 (12.8)
Asian	637 (12.9)	1094 (11.5)	281 (9.5)	432 (9.6)	143 (15.6)
Mixed	822 (16.6)	1779 (18.7)	573 (19.5)	864 (19.2)	124 (13.6)
Other	122 (2.5)	164 (1.7)	65 (2.2)	72 (1.6)	24 (2.6)
Missing	363 (7.4)	510 (5.3)	248 (8.4)	363 (8.1)	60 (6.6)
Smoking status, *n* (%)					
Non-smoker	2434 (49.3)	4967 (52.1)	1416 (48.1)	2031 (45.2)	479 (52.3)
Ex-smoker	1578 (32.0)	3552 (37.3)	1104 (37.5)	1888 (42.0)	300 (32.8)
Current smoker	925 (18.7)	1016 (10.7)	426 (14.5)	577 (12.8)	136 (14.9)

CPRD: Clinical Practice Research Datalink; CTD: connective tissue disease; IIM: idiopathic inflammatory myopathies; IQR: interquartile range; MCTD: mixed connective tissue disease; SjD: Sjögren’s disease; SLE: systemic lupus erythematous; SSc: systemic sclerosis.

### Incidence

**Figure 1.**
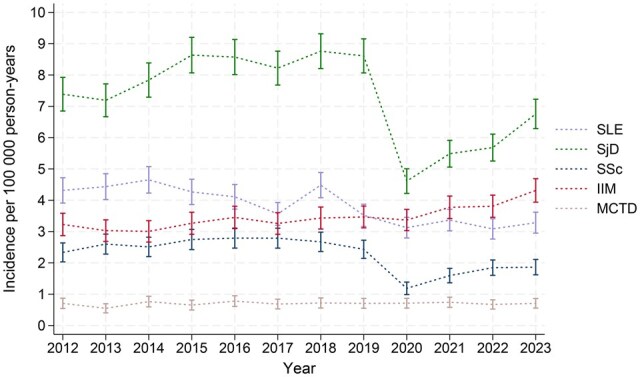
Age and sex standardized incidence rates of SLE, SjD, SSc, IIM and MCTD in CPRD by year, 2012–2023. Rates displayed per 100 000 person-years with 95% CI. CPRD: Clinical Practice Research Datalink; IIM: idiopathic inflammatory myopathies; MCTD: mixed connective tissue disease; SjD: Sjögren’s disease; SLE: systemic lupus erythematous; SSc: systemic sclerosis

Based on CPRD data from England, the age and sex-standardized incidence rate (ASIR) of SLE decreased steadily over the study period (4.32–3.29 per 100 000 person years [py], IRR 0.76, Table 2). In contrast, IIM ASIR rose steadily during the study period (3.23–4.31 per 100 000 py, IRR 1.33), intersecting with the SLE ASIR in 2019 (Fig. 1). Neither SLE nor IIM showed any distinct deviations from underlying trends during the COVID-19 pandemic.

**Table 2.** Age and sex standardized incidence rates (ASIRs) in CPRD by year, 2012–2023, with 95% CI

	SLE		SjD		SSc		IIM		MCTD	
Year	Incident diagnoses, *n*	**ASIR per 100** **000** **py (95% CI)**	Incident diagnoses, *n*	**ASIR per 100** **000** **py (95% CI)**	Incident diagnoses, *n*	**ASIR per 100** **000** **py (95% CI)**	Incident diagnoses, *n*	**ASIR per 100** **000** **py (95% CI)**	Incident diagnoses, *n*	**ASIR per 100** **000** **py (95% CI)**
2012	443	4.32 (3.91, 4.72)	735	7.39 (6.85, 7.92)	232	2.33 (2.03, 2.64)	318	3.23 (2.87, 3.58)	72	0.71 (0.54, 0.87)
2013	450	4.44 (4.02, 4.85)	727	7.19 (6.67, 7.72)	258	2.60 (2.28, 2.92)	302	3.03 (2.69, 3.37)	55	0.55 (0.40, 0.70)
2014	473	4.65 (4.23, 5.07)	797	7.84 (7.29, 8.39)	254	2.51 (2.20, 2.82)	302	3.01 (2.66, 3.35)	79	0.76 (0.59, 0.93)
2015	438	4.27 (3.86, 4.67)	900	8.64 (8.07, 9.20)	282	2.75 (2.42, 3.07)	336	3.27 (2.91, 3.62)	67	0.65 (0.50, 0.81)
2016	428	4.11 (3.72, 4.50)	908	8.57 (8.01, 9.13)	297	2.79 (2.47, 3.11)	363	3.45 (3.09, 3.81)	82	0.78 (0.61, 0.95)
2017	378	3.56 (3.20, 3.93)	892	8.22 (7.68, 8.76)	298	2.79 (2.47, 3.11)	347	3.26 (2.91, 3.60)	75	0.68 (0.53, 0.84)
2018	485	4.48 (4.08, 4.89)	965	8.76 (8.21, 9.32)	290	2.67 (2.36, 2.98)	372	3.43 (3.08, 3.78)	78	0.72 (0.56, 0.88)
2019	385	3.52 (3.16, 3.87)	967	8.61 (8.07, 9.15)	273	2.43 (2.14, 2.72)	385	3.47 (3.12, 3.81)	79	0.71 (0.55, 0.86)
2020	352	3.12 (2.80, 3.45)	530	4.61 (4.22, 5.01)	137	1.19 (0.99, 1.39)	382	3.37 (3.03, 3.71)	81	0.71 (0.56, 0.87)
2021	376	3.37 (3.02, 3.71)	637	5.49 (5.06, 5.91)	184	1.59 (1.36, 1.82)	430	3.77 (3.42, 4.13)	83	0.74 (0.58, 0.90)
2022	353	3.08 (2.76, 3.41)	675	5.68 (5.25, 6.11)	219	1.85 (1.60, 2.09)	447	3.81 (3.46, 4.16)	80	0.67 (0.52, 0.82)
2023	376	3.29 (2.95, 3.62)	802	6.76 (6.29, 7.23)	222	1.86 (1.62, 2.11)	512	4.31 (3.94, 4.69)	84	0.71 (0.56, 0.86)

ASIR: age and sex standardized incidence rate; CPRD: Clinical Practice Research Datalink; IIM: idiopathic inflammatory myopathies; MCTD: mixed connective tissue disease; py: person years; SjD: Sjögren’s disease; SLE: systemic lupus erythematous; SSc: systemic sclerosis.

From 2012 to 2019, SjD incidence rose (ASIR 7.39–8.61 per 100 000 py) but then dropped sharply during the pandemic by 46.5% (Fig. 1) and remained below pre-pandemic levels by 2023 (6.76 per 100 000 py). SSc incidence remained stable from 2012 to 2019 (ASIR 2.33–2.43 per 100 000 py) but also experienced a steep drop during the pandemic (51.0%, Fig. 1), which had not recovered to pre-pandemic levels by 2023 (1.86 per 100 000 py). MCTD ASIR remained stable throughout the study period with no discernible impact from the COVID-19 pandemic (0.71 per 100 000 py in 2012 and 2023).

The fall in SLE and SSc incidence was comparable between males and females (Supplementary Fig. S1), and both sexes showed a rise in IIM incidence. There was a slight divergence in the incidence of SjD and MCTD between males and females, but due to lower relative incidence of these conditions in males, there was little difference overall. More stringent case definitions led to lower incidence estimates across all CTDs, but temporal trends remained consistent (Supplementary Table S2, Fig. S2).

### Mortality

There were 2385 deaths over the whole study period. The median age at death was 75 (IQR 22–98) and was comparable across the studied CTDs (Table 3). The highest age-standardized mortality rate (ASMR) over the study period was seen in the IIM cohort (27.83 per 1000 py, Table 3), followed by SSc (24.43 per 1000 py), SLE (16.74 per 1000 py) and MCTD (16.27 per 1000 py). Individuals with SjD had the lowest relative ASMR (9.70 per 1000 py).

**Table 3.** Number and characteristics of deaths associated within each incident CTD cohort

	SLE	SjD	SSc	IIM	MCTD
	(*n* = 385)	(*n* = 730)	(*n* = 531)	(*n* = 755)	(*n* = 67)
Age at death, median (IQR), years	72.0 (63.0–80.0)	77.0 (69.0–84.0)	75.0 (66.0–82.0)	74.0 (65.0–81.0)	72.0 (60.0–78.0)
Gender, *n* (%)					
Male	94 (24.4)	128 (17.5)	113 (21.3)	395 (52.3)	16 (23.9)
Female	291 (75.6)	602 (82.5)	418 (78.7)	360 (47.7)	51 (76.1)
Ethnicity, *n* (%)					
White	204 (53.0)	361 (49.5)	250 (47.1)	360 (47.7)	31 (46.3)
Black	24 (6.2)	13 (1.8)	17 (3.2)	36 (4.8)	9 (13.4)
Asian	18 (4.7)	45 (6.2)	26 (4.9)	48 (6.4)	10 (14.9)
Mixed and Other	89 (23.1)	203 (27.8)	142 (26.7)	179 (23.7)	*
Missing	50 (13.0)	108 (14.8)	96 (18.1)	132 (17.5)	*
Time from diagnosis to death, median (IQR), years	3.5 (1.5–6.0)	3.8 (1.8–6.2)	2.7 (1.1–5.2)	1.8 (0.6–4.1)	2.7 (0.9–5.8)
Crude mortality rate per 1000 person-years (95% CI)	15.46 (13.92, 17.01)	14.82 (13.74, 15.89)	37.30 (34.13, 40.48)	40.89 (37.97, 43.80)	15.38 (11.70, 19.06)
ASMR per 1000 person-years (95% CI)	16.74 (15.03, 18.46)	9.70 (8.75, 10.65)	24.43 (22.13, 26.72)	27.83 (25.63, 30.03)	16.27 (12.11, 20.42)

Individuals with more than one CTD diagnosis contributed to each relevant group. Counts less than 8 were redacted (*). ASMR: age-standardized mortality rate; CTD: connective tissue disease; IIM: idiopathic inflammatory myopathies; IQR: interquartile range; MCTD: mixed connective tissue disease; SjD: Sjögren’s disease; SLE: systemic lupus erythematous; SSc: systemic sclerosis.

**Figure 2.**
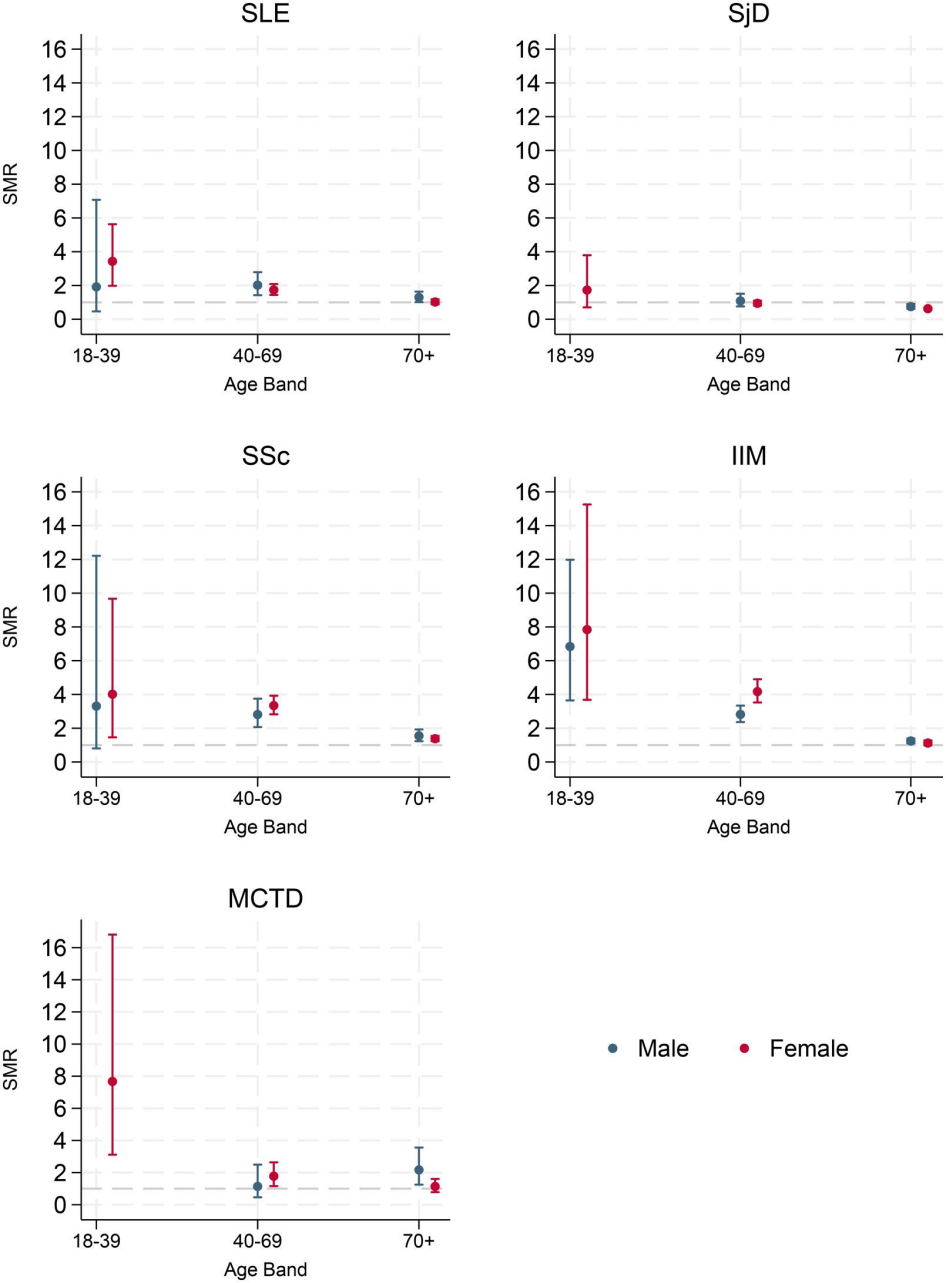
Standardized mortality ratios (SMRs) of SLE, SjD, SSc, IIM and MCTD by age group and sex. SMRs not displayed for males aged 18–39 with SjD or MCTD due to zero events. IIM: idiopathic inflammatory myopathies; MCTD: mixed connective tissue disease; SjD: Sjögren’s disease; SLE: systemic lupus erythematous; SSc: systemic sclerosis

SMRs stratified by age group and sex are displayed in Fig. 2 and Supplementary Table S3. SMRs were highest in the youngest age category (18–39) for all conditions and approached ONS expected death figures for those over 70 years old. The 18–39 year old SMRs were particularly high for IIM (7.21; 95% CI: 4.39, 11.29), MCTD (6.21; 95% CI: 2.52, 13.62), SSc (3.81; 95% CI: 1.55, 8.36) and SLE (3.23; 95% CI: 1.90, 5.22), although confidence intervals were wide due to low numbers of total events. There were no male deaths in the 18–39-year-old category for those with MCTD or SjD, which likely reflected the lower incidence of these conditions in young males, so a SMR was not calculated. Overall, males had higher SMRs than females for SLE, SjD, SSc and MCTD, whilst the SMRs for the IIM group were more even across sexes (male SMR 1.54 *vs* female SMR 1.58).

**Figure 3.**
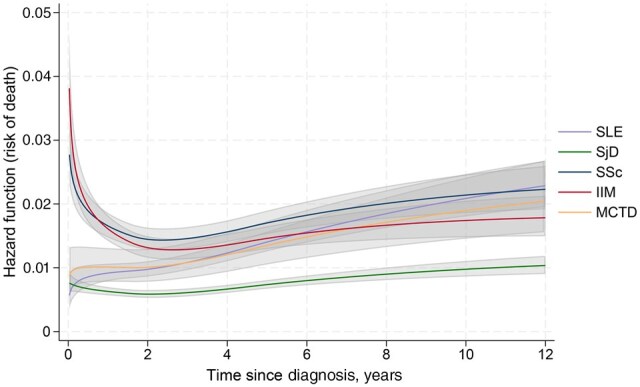
Risk of death following diagnosis of SLE, SjD, SSc, IIM and MCTD over time. Hazard functions modelled using flexible parametric curves with restricted cubic splines; 95% confidence intervals shown as shaded areas. IIM: idiopathic inflammatory myopathies; MCTD: mixed connective tissue disease; SjD: Sjögren’s disease; SLE: systemic lupus erythematous; SSc: systemic sclerosis

The highest risk of death in relation to time from diagnosis was seen in those with IIM, closely followed by those diagnosed with SSc (Fig. 3). The risk of death fell steeply for IIM, in line with SSc at approximately 1 year, after which both reached a nadir at 2 years from diagnosis. The risk of death at diagnosis was similar across individuals with SLE, MCTD and SjD, but rose for those with SLE and MCTD over time intersecting the risk of death for those with IIM at approximately 6 years. The risk of death from SjD diagnosis was persistently low in comparison with the other CTDs.

Sensitivity analyses exploring the effect of multiple CTD diagnoses and the COVID-19 pandemic were conducted for mortality. ASMRs showed minimal changes when those with multiple CTD diagnoses were excluded (Supplementary Table S4). SMR values were also generally equivalent after exclusion of those with multiple CTD diagnoses, except the SMR for males with MCTD, which fell by 16.4% (Supplementary Table S5). Investigating the impact of the COVID-19 pandemic on mortality, ASMRs increased for all CTDs except SjD in the 2020–2023 period compared with 2012–2019 (Supplementary Tables S6 and S7). Corresponding SMRs for incident CTD cases also rose, suggestive of deaths in excess of those seen in the general English population (Supplementary Tables S8 and S9).

## Discussion

In this large population-level cohort we reported a falling incidence of SLE, a rising incidence of IIM, and high mortality for IIM and SSc relative to other CTDs. Our description of a large English incident cohort provides vital updates to the epidemiological structure of CTDs in the modern biologic therapy era.

In general, the incidence of individual CTDs in England is inconsistently reported and dependent upon the methodological approach used. This is compounded by superseded classification criteria, increased awareness of rare conditions and advancing technology over time in terms of imaging and immunoassays (e.g. myositis-specific antibodies). This could explain the observed falling incidence of SLE and rising incidence of IIM, whereby those who previously may have been diagnosed with SLE may now be diagnosed with IIM in the context of more specific investigations.

In keeping with our findings and using similar methodology, Rees *et al.* described a fall in SLE incidence from 1999 to 2012 (5.10 to 4.64 per 100 000 py) using a separate CPRD dataset (GOLD) [10]. Notably, our SLE cohort included relatively greater racial and ethnic diversity with less missing ethnicity data. More recently, Conrad *et al.* utilized both CPRD GOLD and Aurum datasets to demonstrate higher ASIRs than we observed for SLE, SjD and SSc (9.4, 10.7 and 3.3 per 100 000 py in 2017–2019, respectively) [5]. They also saw a rising incidence for all three conditions from 2000–2002 to 2017–2019. However, within a sensitivity analysis with more restrictive definitions, they described a lower incidence of SLE, SjD and SSc in 2017–2019 (5.7, 10.3 and 2.9 per 100 000 py, respectively). Additionally, by excluding antiphospholipid syndrome, cutaneous, discoid and drug-induced lupus, they reported a fall in the incidence of SLE over time (IRR 0.93). Other UK-based studies revealed comparable results to our observed SLE [10, 13] and SSc incidence [14], whilst other reports showed a lower SSc incidence [8, 15], highlighting said inconsistencies.

IIM was largely thought to be a rare condition with three reports of its incidence in England varying from 1.30 to 1.76 per 100 000 py between 2000 and 2019 [6, 16, 17]. In contrast, our results demonstrated a much higher incidence of IIM, possibly due to our large primary care dataset, differing definitions, or potential misclassification or ascertainment biases. To our knowledge, there were no peer-reviewed studies describing MCTD incidence in the UK.

We decided to use a single code strategy for case ascertainment *a priori* in line with other key studies [5, 10]. However, we recognized alternative definitions and strategies could affect absolute incidence rates and ran a sensitivity analysis using a more stringent definition for identifying incident cases (Supplementary Table S2 and Fig. S2). As expected, absolute ASIRs were reduced for all CTDs, particularly SjD, SSc and IIM, but importantly relative trends were preserved.

The COVID-19 pandemic affected medical services worldwide, with a well-described drop in the incidence of inflammatory arthritis across England [18]. Our study showed a 46.4% fall in SjD diagnoses and 51.2% fall in SSc diagnoses compared with a 20.3% reduction in inflammatory arthritis over the same period [18]. In contrast, the incidence of MCTD, SLE and IIM was less impacted by the COVID-19 pandemic. In general, SjD is considered a more indolent condition compared to the other CTDs and would have been less likely to present during the pandemic given the diversion of attention and resources to acute healthcare. The drop in SSc incidence during the pandemic could be explained by its relative rarity compared with other CTDs, an associated lack of awareness or a lower perceived urgency of limited disease. Similar to Russell *et al.*’s findings in inflammatory arthritis [18], we saw no rebound increase in the incidence of SjD or SSc indicating a potential group of individuals with undiagnosed SjD and SSc in the population.

Mortality data displayed two salient features. Firstly, IIM, SSc, SLE and MCTD were all associated with high mortality. Deaths were most likely due to the multi-systemic nature of these conditions, which commonly involve cardiovascular, renal and respiratory complications as well as a frequent use of immunosuppressive therapy and increased susceptibility to infections [19]. IIM and SSc were associated with a particularly high risk of death early after diagnosis, which may be representative of strong associations with malignancy for IIM [20], pulmonary hypertension for SSc [21] and interstitial lung disease for both [22].

MCTD remains a contentious diagnosis among rheumatologists [26] with prominent overlap with other CTDs. The surprisingly high mortality associated with MCTD could be explained by diagnostic overlay with other CTDs that had not clearly differentiated themselves or did so prior to death and re-coding.

Secondly, the youngest age group (18–39 years) showed the highest excess death for all conditions compared with ONS data, although we noted low numbers of absolute deaths for this group (13 SLE, 15 IIM and less than eight SjD, SSc and MCTD deaths) (Supplementary Table S3). This was anticipated as CTDs are commonly diagnosed in younger individuals, an age group that otherwise has relatively low baseline mortality. Despite fewer overall deaths, the excess mortality in our youngest patients with CTD could be attributed to late recognition, reduced awareness, poor health literacy, inadequate advocacy or aggressive disease [23–25]. Regardless, this observation highlighted an at-risk subset of individuals within an already vulnerable population.

Focusing on the impact of the COVID-19 pandemic on mortality, ASMRs and SMRs increased for all CTDs except SjD in the 2020–2023 period compared with 2012–2019 (Supplementary Tables S5, S6, S8 and S9). This was an unsurprising finding given that CTDs often present in younger individuals and are treated with immunosuppression [5, 10, 27]. Exclusion of multiple CTD diagnoses led to little change in calculated ASMRs and SMRs (Supplementary Tables S4 and S7).

We observed a crude mortality rate of 15.46 per 1000 py in the SLE cohort, which was consistent with previous CPRD reports for SLE (15.84 per 1000 py) [9]. Although Rees *et al.* [9] did not publish ASMRs or SMRs for direct comparison, we noted similar trends with higher mortality in younger groups with SLE. Previous English studies reported crude mortality rates of 34.4 and 49.1 per 1000 py for polymyositis and dermatomyositis, respectively (1999–2014) [7], which was equivalent to our observed IIM crude mortality rate of 40.89 per 1000 py. Using CPRD, Pauling *et al.* identified a SSc cohort with a mean mortality rate of 32 per 1000 py between 1998 and 2017 [8], which was similar to our crude mortality rate of 37.30 per 1000 py. However, neither study explicitly commented on ASMRs, making direct comparisons difficult.

No studies summarizing comparable statistics of mortality in England for SjD or MCTD were found. In stark contrast to our findings, a meta-analysis of 14 international SjD studies showed a SMR of 1.46, although heterogeneity was high (*I*^2^ 92.4%) [28]. Importantly, all included European studies (*n* = 9) started in the 1970s–1980s with the latest ending in 2010, making this pooled cohort incomparable to ours.

This study had several strengths. First, the mortality of CTDs had not been examined utilizing the CPRD Aurum dataset before. This was crucial to our study, given the significant progress in classifications, investigations and management of CTDs in recent years. Second, the large population size and broad coverage of the Aurum dataset meant we could identify sizable individual CTD cohorts and examine temporal trends. Third, we described and compared the incidence and mortality of multiple CTDs in one report, which is of significant value to clinicians and health resource planning. Fourth, to allow comparison across studies and countries we standardized data to the ESP and reported multiple mortality descriptors (ASMR, SMR and risk).

Diagnostic miscoding in primary care records may have led to over- or under-estimation of CTD incidence. To minimize misclassification, our code lists were scrutinized by three members of the team and were directly compared with other CPRD studies [5, 6, 10]. Although we required 12 months prior registration to minimize misclassification, some prevalent cases may have been incorrectly classified as incident. Similarly, while we could not exclude all pre-existing cases from denominator calculations, the rarity of CTDs and the large population size suggest any resulting bias was likely minimal.

We were unable to determine further information such as disease severity and organ involvement, as these were mostly reviewed and documented in secondary care, limiting our inference of disease phenotype with mortality. Also, with the possibility of co-occurrence amongst CTDs, the mortality of individual CTDs may have been inaccurately estimated in those with multiple diagnoses. To address this, we conducted a sensitivity analysis and excluded those with more than one CTD diagnosis where no major differences were seen in the ASMR or SMR across the studied CTDs (Supplementary Tables S4 and S7). Finally, our study period included the COVID-19 pandemic, which affected mortality data, so we also summarized statistics before and after pandemic onset (Supplementary Tables S5, S6, S8 and S9).

## Conclusions

We provided contemporary epidemiological data on the incidence and mortality across a spectrum of CTDs in England. The patterns of CTDs are evolving, with a rising incidence of IIM and a commensurate decline in the incidence of SLE, more likely driven by changing disease classification criteria than true shifts in disease epidemiology. Our findings on mortality underscore the significant burden these diseases impose, particularly on younger patients and those with IIM or SSc. These results are essential for healthcare resource planning and highlight the importance of early diagnosis to improve outcomes for this vulnerable population.

## Supplementary Material

keaf414_Supplementary_Data

## Data Availability

All data relevant to the study are included in the article or uploaded as supplementary information.
