# Supplementary material for: The incidence and mortality of connective tissue diseases: a population-level cohort study in England from 2012 to 2023
Source: Rheumatology (Oxford). 2025 Aug 4;64(12):6151–8. doi: 10.1093/rheumatology/keaf414 (PMC12671860; doi:10.1093/rheumatology/keaf414)
Supplement: keaf414_Supplementary_Data [file keaf414_supplementary_data.docx]

**Supplementary material**

[Supplementary table S1. SNOMED-CT code lists for SLE, MCTD, SjD, SSc and IIM. 2](#_Toc203121922)

[Supplementary table S2. Age and sex standardised incidence rates (ASIRs) in CPRD using restricted definitions by year, 2012-2023, with 95% confidence intervals (95% CI) 8](#_Toc203121923)

[Supplementary table S3. Number of observed deaths, expected deaths and standardised mortality ratios over the study period by CTD, ag group and sex. Counts less than eight were redacted (*). 9](#_Toc203121924)

[Supplementary table S4. Age and sex standardised mortality rates for incident CTD diagnoses excluding cases with more than one CTD diagnosis (before or during our study period). 10](#_Toc203121925)

[Supplementary table S5. Standardised mortality ratios (SMRs) excluding cases with more than one CTD diagnosis. Displayed by CTD and sex. 11](#_Toc203121926)

[Supplementary table S6. Age and sex standardised mortality rates for incident CTD diagnoses from 2012 to 2019 (pre-COVID-19 pandemic). 12](#_Toc203121927)

[Supplementary table S7. Age and sex standardised mortality rates for incident CTD diagnoses from 2020 to 2023 (post-COVID-19 pandemic). 13](#_Toc203121928)

[Supplementary table S8. Standardised mortality ratios (SMRs) from 2012 – 2019 (pre-COVID-19 pandemic). Displayed by CTD and sex. Counts less than eight were redacted (*). 14](#_Toc203121929)

[Supplementary table S9. Standardised mortality ratios (SMRs) from 2020 - 2023 (post-COVID-19 pandemic). Displayed by CTD and sex. Counts less than eight were redacted (*). 15](#_Toc203121930)

[Supplementary figure S1. Individual graphs of age standardised incidence rates (ASIRs) by sex, with 95% confidence intervals (95% CI). 1a. SLE, 1b. SjD, 1c. SSc, 1d. IIM, 1e. MCTD. 16](#_Toc203121931)

[Supplementary figure S2. Age and sex standardised incidence rates (ASIRs) using restricted definitions in CPRD by year, 2012-2023, with 95% confidence intervals (95% CI). 19](#_Toc203121932)

# Supplementary table S1. SNOMED-CT code lists for SLE, MCTD, SjD, SSc and IIM.

| Diagnostic code | SNOMED CT Concept ID | Term |
| --- | --- | --- |
| 312579010 | 55464009 | [X]Other forms of systemic lupus erythematosus |
| 6681821000006113 | 403486000 | Acute systemic lupus erythematosus |
| 5140891000006113 | 239889005 | Bullous systemic lupus erythematosus |
| 4054931000006112 | 95332009 | Butterfly rash associated with systemic lupus |
| 6682071000006111 | 403505001 | Calcinosis secondary to lupus erythematosus |
| 512234017 | 95644001 | Cerebral lupus |
| 4060291000006118 | 95644001 | Cerebral systemic lupus erythematosus |
| 5006111000006112 | 230307005 | Chorea in systemic lupus erythematosus |
| 7860991000006115 | 724767000 | Chorea with systemic lupus erythematosus |
| 7861201000006113 | 724781003 | Demyelination of central nervous system co-occurrent and due to systemic lupus erythematosus |
| 7861211000006111 | 724781003 | Demyelination with systemic lupus erythematosus |
| 3087841000006114 | 36471000 | Dilated cardiomyopathy secondary to systemic lupus erythematosus |
| 8022621000006111 | 72181000119109 | Endocarditis due to systemic lupus erythematosus |
| 6681831000006111 | 403487009 | Fulminating systemic lupus erythematosus |
| 8099911000006113 | 308751000119106 | Glomerular disease due to SLE (systemic lupus erythematosus) |
| 8099901000006110 | 308751000119106 | Glomerular disease due to systemic lupus erythematosus |
| 7951841000006111 | 732960002 | Haemolytic anaemia associated with systemic lupus erythematosus |
| 7951851000006113 | 732960002 | Hemolytic anemia associated with systemic lupus erythematosus |
| 301721015 | 196138005 | Lung disease with systemic lupus erythematosus |
| 3765031000006115 | 77753005 | Lupus disease of the lung |
| 4060271000006119 | 95644001 | Lupus encephalopathy |
| 6682151000006119 | 403511003 | Lupus erythematosus-associated necrotising vasculitis |
| 6682161000006117 | 403511003 | Lupus erythematosus-associated necrotizing vasculitis |
| 5141751000006119 | 239944008 | Lupus erythematosus-associated vasculitis |
| 2812511000006115 | 19682006 | Lupus hepatitis |
| 114310015 | 68815009 | Lupus nephritis |
| 3691781000006113 | 73286009 | Lupus nephritis - WHO Class I |
| 2573601000006117 | 4676006 | Lupus nephritis - WHO Class II |
| 3744661000006111 | 76521009 | Lupus nephritis - WHO Class III |
| 3086691000006118 | 36402006 | Lupus nephritis - WHO Class IV |
| 3343601000006112 | 52042003 | Lupus nephritis - WHO Class V |
| 2675171000006110 | 11013005 | Lupus nephritis - WHO Class VI |
| 5055001000006112 | 233730002 | Lupus pneumonia |
| 5141731000006114 | 239944008 | Lupus vasculitis |
| 2675161000006115 | 11013005 | Lupus with glomerular sclerosis |
| 3343591000006116 | 52042003 | Membranous lupus glomerulonephritis |
| 297639014 | 193248005 | Myopathy due to disseminated lupus erythematosus |
| 6682171000006112 | 403511003 | Necrotising vasculitis due to lupus erythematosus |
| 6682181000006110 | 403511003 | Necrotizing vasculitis due to lupus erythematosus |
| 8091131000006116 | 295101000119105 | Nephropathy co-occurrent and due to systemic lupus erythematosus |
| 8091161000006113 | 295121000119101 | Nephrosis co-occurrent and due to systemic lupus erythematosus |
| 8091171000006118 | 295121000119101 | Nephrosis with systemic lupus erythematosus |
| 8091151000006111 | 295111000119108 | Nephrotic syndrome co-occurrent and due to systemic lupus erythematosus |
| 677561000006119 | 68815009 | Nephrotic syndrome in systemic lupus erythematosus |
| 11851321000006119 | 25380002 | Pericarditis co-occurrent and due to systemic lupus erythematosus |
| 2905941000006111 | 25380002 | Pericarditis secondary to systemic lupus erythematosus |
| 297542011 | 193178008 | Polyneuropathy in disseminated lupus erythematosus |
| 4054921000006114 | 95332009 | Rash of systemic lupus erythematosus |
| 177301000006114 | 307755009 | Renal tubulo-interstitial disorder in systemic lupus erythematosus |
| 6905051000006113 | 417303004 | Retinal vasculitis due to systemic lupus erythematosus |
| 7951821000006116 | 732960002 | Secondary autoimmune haemolytic anaemia co-occurrent and due to systemic lupus erythematosus |
| 7951831000006118 | 732960002 | Secondary autoimmune hemolytic anemia co-occurrent and due to systemic lupus erythematosus |
| 5140861000006117 | 239886003 | Skin and joint lupus |
| 3400601000006117 | 55464009 | SLE - Systemic lupus erythematosus |
| 6593821000006117 | 397856003 | SLE - Systemic lupus erythematosus-related syndrome |
| 3619221000006118 | 68815009 | SLE glomerulonephritis syndrome |
| 3691761000006115 | 73286009 | SLE glomerulonephritis syndrome, WHO class I |
| 2573581000006110 | 4676006 | SLE glomerulonephritis syndrome, WHO class II |
| 3744641000006112 | 76521009 | SLE glomerulonephritis syndrome, WHO class III |
| 3086671000006119 | 36402006 | SLE glomerulonephritis syndrome, WHO class IV |
| 3343571000006117 | 52042003 | SLE glomerulonephritis syndrome, WHO class V |
| 2675141000006119 | 11013005 | SLE glomerulonephritis syndrome, WHO class VI |
| 2675151000006117 | 11013005 | SLE with advanced sclerosing glomerulonephritis |
| 3086681000006116 | 36402006 | SLE with diffuse proliferative glomerulonephritis |
| 3744651000006114 | 76521009 | SLE with focal AND segmental proliferative glomerulonephritis |
| 3343581000006119 | 52042003 | SLE with membranous glomerulonephritis |
| 2573591000006113 | 4676006 | SLE with mesangial proliferative glomerulonephritis |
| 3691771000006110 | 73286009 | SLE with normal kidneys |
| 92208011 | 55464009 | Systemic lupus erythematosus |
| 4056181000006118 | 95408003 | Systemic lupus erythematosus arthritis |
| 1728151000006118 | 1728151000006102 | Systemic lupus erythematosus encephalitis |
| 158442019 | 95644001 | Systemic lupus erythematosus encephalitis |
| 3619241000006113 | 68815009 | Systemic lupus erythematosus glomerulonephritis syndrome |
| 3691791000006111 | 73286009 | Systemic lupus erythematosus glomerulonephritis syndrome, World Health Organisation (WHO) class I |
| 2573611000006119 | 4676006 | Systemic lupus erythematosus glomerulonephritis syndrome, World Health Organisation (WHO) class II |
| 3744681000006118 | 76521009 | Systemic lupus erythematosus glomerulonephritis syndrome, World Health Organisation (WHO) class III |
| 3086701000006118 | 36402006 | Systemic lupus erythematosus glomerulonephritis syndrome, World Health Organisation (WHO) class IV |
| 3343611000006110 | 52042003 | Systemic lupus erythematosus glomerulonephritis syndrome, World Health Organisation (WHO) class V |
| 2675181000006113 | 11013005 | Systemic lupus erythematosus glomerulonephritis syndrome, World Health Organisation (WHO) class VI |
| 3691801000006112 | 73286009 | Systemic lupus erythematosus glomerulonephritis syndrome, World Health Organization (WHO) class I |
| 2573621000006110 | 4676006 | Systemic lupus erythematosus glomerulonephritis syndrome, World Health Organization (WHO) class II |
| 3744671000006116 | 76521009 | Systemic lupus erythematosus glomerulonephritis syndrome, World Health Organization (WHO) class III |
| 3086711000006115 | 36402006 | Systemic lupus erythematosus glomerulonephritis syndrome, World Health Organization (WHO) class IV |
| 3343621000006119 | 52042003 | Systemic lupus erythematosus glomerulonephritis syndrome, World Health Organization (WHO) class V |
| 2675191000006111 | 11013005 | Systemic lupus erythematosus glomerulonephritis syndrome, World Health Organization (WHO) class VI |
| 3691811000006110 | 73286009 | Systemic lupus erythematosus glomerulonephritis syndrome, World Health Organization class I |
| 2573631000006113 | 4676006 | Systemic lupus erythematosus glomerulonephritis syndrome, World Health Organization class II |
| 3744691000006115 | 76521009 | Systemic lupus erythematosus glomerulonephritis syndrome, World Health Organization class III |
| 3086721000006111 | 36402006 | Systemic lupus erythematosus glomerulonephritis syndrome, World Health Organization class IV |
| 3343631000006116 | 52042003 | Systemic lupus erythematosus glomerulonephritis syndrome, World Health Organization class V |
| 2675201000006114 | 11013005 | Systemic lupus erythematosus glomerulonephritis syndrome, World Health Organization class VI |
| 7511501000006112 | 698694005 | Systemic lupus erythematosus in remission |
| 309408013 | 55464009 | Systemic lupus erythematosus NOS |
| 5140901000006112 | 239890001 | Systemic lupus erythematosus with multisystem involvement |
| 114251000006118 | 239887007 | Systemic lupus erythematosus with organ/system involvement |
| 453243010 | 309762007 | Systemic lupus erythematosus with pericarditis |
| 8320771000006119 | 905381000000108 | Systemic lupus erythematosus/Sjogren's overlap syndrome |
| 6670991000006115 | 402865003 | Systemic lupus erythematosus-associated antiphospholipid syndrome |
| 6593811000006113 | 397856003 | Systemic lupus erythematosus-related syndrome |
| 5141741000006116 | 239944008 | Vasculitis due to lupus erythematosus |
| 7861221000006115 | 724782005 | Demyelination of central nervous system co-occurrent and due to Sjogren disease |
| 7861231000006117 | 724782005 | Demyelination of central nervous system with Sjogren disease |
| 3783951000006114 | 78946008 | Keratoconjunctivitis sicca, in Sjögren syndrome |
| 3783931000006119 | 78946008 | Keratoconjunctivitis sicca, in Sjögren's syndrome |
| 3783941000006112 | 78946008 | Keratoconjunctivitis sicca, in Sjogren's syndrome |
| 4782481000006115 | 196137000 | Lung disease with Sjögren disease |
| 4782461000006113 | 196137000 | Lung disease with Sjögren's disease |
| 301720019 | 196137000 | Lung disease with Sjogren's disease |
| 4386931000006110 | 126766000 | Lymphoepithelial sialadenitis of Sjögren syndrome |
| 4386901000006119 | 126766000 | Lymphoepithelial sialadenitis of Sjögren's syndrome |
| 4386921000006112 | 126766000 | Lymphoepithelial sialadenitis of Sjogren's syndrome |
| 4386911000006116 | 126766000 | Myoepithelial sialadenitis in Sjögren's syndrome |
| 4770991000006115 | 193253000 | Myopathy due to Sjögren disease |
| 4770971000006116 | 193253000 | Myopathy due to Sjögren's disease |
| 4771001000006119 | 193253000 | Myopathy due to Sjogrens disease |
| 297645018 | 193253000 | Myopathy due to Sjogren's disease |
| 5141131000006113 | 239912009 | Primary Sjögren syndrome |
| 5141191000006112 | 239914005 | Primary Sjögren syndrome with multisystem involvement |
| 5141161000006116 | 239913004 | Primary Sjögren syndrome with organ/system involvement |
| 5141111000006119 | 239912009 | Primary Sjögren's syndrome |
| 5141171000006111 | 239914005 | Primary Sjögren's syndrome with multisystem involvement |
| 5141141000006115 | 239913004 | Primary Sjögren's syndrome with organ/system involvement |
| 5141121000006110 | 239912009 | Primary Sjogren's syndrome |
| 5141181000006114 | 239914005 | Primary Sjogren's syndrome with multisystem involvement |
| 5141151000006118 | 239913004 | Primary Sjogren's syndrome with organ/system involvement |
| 3223221000006113 | 44833003 | Pseudolymphoma of lung in Sjögren disease |
| 3223201000006115 | 44833003 | Pseudolymphoma of lung in Sjögren's disease |
| 3223211000006117 | 44833003 | Pseudolymphoma of lung in Sjogren's disease |
| 141091000006112 | 83901003 | Sicca syndrome |
| 3865071000006119 | 83901003 | Sjögren syndrome |
| 3865051000006112 | 83901003 | Sjögren's disease |
| 3865031000006117 | 83901003 | Sjögren's syndrome |
| 3865081000006116 | 83901003 | Sjogrens syndrome |
| 3865061000006114 | 83901003 | Sjogren's syndrome |
| 8102691000006113 | 320681000119102 | Tubulointerstitial nephropathy due to Sjögren's syndrome |
| 8102681000006110 | 320681000119102 | Tubulointerstitial nephropathy due to Sjogren syndrome |
| 8102701000006113 | 320681000119102 | Tubulointerstitial nephropathy due to Sjogrens syndrome |
| 312581012 | 89155008 | [X]Other forms of systemic sclerosis |
| 354513019 | 236503001 | Acute scleroderma renal crisis |
| 6682241000006113 | 403514006 | Calcinosis cutis due to systemic sclerosis |
| 3012911000006115 | 31848007 | Calcinosis cutis, Raynaud's, esophageal dysfunction, sclerodactyly AND telangiectasia |
| 3012921000006111 | 31848007 | Calcinosis cutis, Raynaud's, oesophageal dysfunction, sclerodactyly AND telangiectasia |
| 3514021000006117 | 62382002 | Calcinosis cutis, Raynaud's, sclerodactyly AND telangiectasia |
| 3012991000006113 | 31848007 | Calcinosis, Raynaud phenomenon, esophageal dysmotility, sclerodactyly, and telangiectasia (CREST) syndrome |
| 3514061000006111 | 62382002 | Calcinosis, Raynaud phenomenon, sclerodactyly, and telangiectasia (CRST) syndrome |
| 3012981000006110 | 31848007 | Calcinosis, Raynaud's phenomenon, esophageal dysmotility, sclerodactyly, and telangiectasia (CREST) syndrome |
| 3013001000006114 | 31848007 | Calcinosis, Raynaud's phenomenon, esophageal dysmotility, sclerodactyly, and telangiectasia syndrome |
| 3012971000006112 | 31848007 | Calcinosis, Raynaud's phenomenon, oesophageal dysmotility, sclerodactyly, and telangiectasia (CREST) syndrome |
| 3514051000006114 | 62382002 | Calcinosis, Raynaud's phenomenon, sclerodactyly, and telangiectasia (CRST) syndrome |
| 3514071000006116 | 62382002 | Calcinosis, Raynaud's phenomenon, sclerodactyly, and telangiectasia syndrome |
| 3514041000006112 | 62382002 | Calcinosis, Raynaud's phenomenon, sclerodactyly, telangiectasia syndrome |
| 3012951000006119 | 31848007 | CREST - Calcinosis, Raynaud's phenomenon, esophageal dysfunction, sclerodactyly, telangiectasia |
| 3012931000006114 | 31848007 | CREST - Calcinosis, Raynaud's phenomenon, oesophageal dysfunction, sclerodactyly, telangiectasia |
| 3012961000006117 | 31848007 | CREST - Calcinosis, Raynaud's phenomenon, sclerodactyly, esophageal involvement, telangiectasia syndrome |
| 3012941000006116 | 31848007 | CREST - Calcinosis, Raynaud's phenomenon, sclerodactyly, oesophageal involvement, telangiectasia syndrome |
| 3514031000006119 | 62382002 | CRST - Calcinosis, Raynaud's phenomenon, sclerodactyly, telangiectasia syndrome |
| 6669081000006115 | 402713007 | Cutaneous complication of systemic sclerosis |
| 4409391000006114 | 128460000 | Diffuse cutaneous scleroderma |
| 4409381000006111 | 128460000 | Diffuse cutaneous systemic sclerosis |
| 4409371000006113 | 128460000 | Diffuse scleroderma |
| 4409361000006118 | 128460000 | Diffuse systemic sclerosis |
| 3075181000006114 | 35719004 | Dilated cardiomyopathy due to systemic sclerosis |
| 3075191000006112 | 35719004 | Dilated cardiomyopathy secondary to scleroderma |
| 2619401000006115 | 7513007 | Generalised scleroderma |
| 2619411000006117 | 7513007 | Generalized scleroderma |
| 8027031000006117 | 89681000119101 | Glomerulonephritis co-occurrent and due to scleroderma |
| 5850811000006111 | 298285004 | Limited cutaneous systemic sclerosis |
| 5864721000006113 | 299276009 | Limited scleroderma |
| 5864711000006117 | 299276009 | Limited systemic sclerosis |
| 301716018 | 196133001 | Lung disease with systemic sclerosis |
| 297644019 | 193252005 | Myopathy due to scleroderma |
| 4770951000006114 | 193252005 | Myopathy due to systemic sclerosis |
| 6682391000006110 | 403520007 | Nail dystrophy due to systemic sclerosis |
| 6682331000006111 | 403518009 | Necrotising vasculitis due to scleroderma |
| 6682341000006118 | 403518009 | Necrotizing vasculitis due to scleroderma |
| 11814811000006110 | 87442008 | Pericarditis co-occurrent and due to scleroderma |
| 3921451000006115 | 87442008 | Pericarditis due to systemic sclerosis |
| 3921461000006118 | 87442008 | Pericarditis secondary to scleroderma |
| 6682361000006119 | 403519001 | Poikiloderma due to systemic sclerosis |
| 8103261000006110 | 322461000119108 | Polyneuropathy due to systemic sclerosis |
| 7731531000006110 | 715401008 | Primary biliary cirrhosis co-occurrent with systemic scleroderma |
| 147834015 | 444133002 | Progressive systemic sclerosis |
| 7288571000006116 | 444133002 | PSS - Progressive systemic sclerosis |
| 354512012 | 236502006 | Renal involvement in scleroderma |
| 6995961000006113 | 422801000 | Scleroderma |
| 309413012 | 89155008 | Scleroderma |
| 4782401000006112 | 196133001 | Scleroderma lung disease |
| 4782411000006110 | 196133001 | Scleroderma of lung |
| 3949121000006118 | 89155008 | Scleroderma syndrome |
| 5141061000006115 | 239903007 | Scleroderma with multisystem involvement |
| 5141051000006117 | 239902002 | Scleroderma with organ / system involvement |
| 6682231000006115 | 403514006 | Scleroderma-associated calcinosis |
| 6682381000006112 | 403520007 | Scleroderma-associated nail dystrophy |
| 6682291000006116 | 403517004 | Scleroderma-associated nailfold telangiectasia |
| 6682311000006117 | 403518009 | Scleroderma-associated necrotising vasculitis |
| 6682321000006113 | 403518009 | Scleroderma-associated necrotizing vasculitis |
| 6682351000006116 | 403519001 | Scleroderma-associated poikiloderma |
| 6682271000006117 | 403516008 | Scleroderma-associated telangiectasia |
| 3949131000006115 | 89155008 | SS - Systemic sclerosis |
| 3949101000006111 | 89155008 | Systemic scleroderma |
| 147833014 | 89155008 | Systemic sclerosis |
| 4409411000006114 | 128461001 | Systemic sclerosis sine scleroderma |
| 5850801000006113 | 298285004 | Systemic sclerosis with limited cutaneous involvement |
| 4409421000006118 | 128461001 | Systemic sclerosis without skin thickening |
| 4409401000006111 | 128460000 | Systemic sclerosis, diffuse |
| 5864731000006111 | 299276009 | Systemic sclerosis, limited |
| 6682281000006119 | 403516008 | Telangiectasia due to systemic sclerosis |
| 6682301000006115 | 403517004 | Telangiectasia of nailfolds due to systemic sclerosis |
| 376791000006111 | 239901009 | [X]Dermato(poly)myositis in neoplastic disease CE |
| 312591018 | 396230008 | [X]Dermatopolymyositis, unspecified |
| 12466821000006119 | 419921000000105 | [X]Dermatopolymyositis, unspecified |
| 299392018 | 128496001 | [X]Inflammatory myopathy, not elsewhere classified |
| 312580013 | 396230008 | [X]Other dermatomyositis |
| 6665201000006115 | 402425006 | Adult onset dermatomyositis |
| 5128191000006115 | 238935002 | Amyopathic dermatomyositis |
| 1166881000000114 | 445187004 | Antisynthetase syndrome |
| 7284931000006119 | 443899007 | Autoimmune inflammation of skeletal muscle |
| 7838981000006111 | 722991004 | Autoimmune myopathy |
| 7284941000006112 | 443899007 | Autoimmune myositis |
| 7738851000006114 | 715863001 | Autoimmune necrotising myopathy |
| 7738861000006111 | 715863001 | Autoimmune necrotizing myopathy |
| 1776211019 | 396230008 | Dermatomyositis |
| 5128181000006118 | 238935002 | Dermatomyositis sine myositis |
| 5141031000006112 | 239901009 | Dermatomyositis with malignant disease |
| 359459016 | 239901009 | Dermatopolymyositis in neoplastic disease |
| 614691000006113 | 396230008 | Dermatopolymyositis, unspecified |
| 3845831000006116 | 82725007 | Diffuse progressive ossifying polymyositis |
| 3254291000006112 | 46696008 | Dilated cardiomyopathy secondary to dermatomyositis |
| 8092571000006119 | 296241000119107 | Disorder of respiratory system due to dermatomyositis |
| 6566141000006112 | 396230008 | DM - Dermatomyositis |
| 5650531000006113 | 281358000 | Idiopathic dermatomyositis |
| 7559361000006114 | 702380008 | Idiopathic inflammatory myopathy |
| 7559371000006119 | 702380008 | Idiopathic inflammatory myositis |
| 5650521000006110 | 281357005 | Idiopathic polymyositis |
| 7738881000006118 | 715863001 | Immune mediated necrotising myopathy |
| 7738871000006116 | 715863001 | Immune mediated necrotizing myopathy |
| 4410181000006116 | 128496001 | Inflammatory myopathy |
| 12047181000006112 | 766706007 | Inflammatory myopathy with abundant macrophages |
| 4782441000006114 | 196136009 | Lung disease co-occurrent with polymyositis |
| 301719013 | 196136009 | Lung disease with polymyositis |
| 311701010 | 26889001 | Myositis |
| 3350911000006111 | 52486002 | Necrotising myositis |
| 3350901000006113 | 52486002 | Necrotizing myositis |
| 7265011000006111 | 442486007 | Paraneoplastic myositis |
| 3005751000006112 | 31384009 | PM - Polymyositis |
| 143230018 | 396230008 | Poikilodermatomyositis |
| 3904271000006115 | 86365006 | Poikilodermatomyositis |
| 52463013 | 31384009 | Polymyositis |
| 5141011000006118 | 239899000 | Polymyositis associated with autoimmune disease |
| 5141001000006116 | 239898008 | Polymyositis with malignant disease |
| 4782451000006111 | 196136009 | Polymyositis with pulmonary involvement |
| 6566131000006119 | 396230008 | Polymyositis with skin involvement |
| 8012461000006114 | 24861000119103 | Symptomatic inflammatory myopathy |
| 4770871000006110 | 193246009 | Symptomatic inflammatory myopathy associated with another disorder |
| 297637011 | 193246009 | Symptomatic inflammatory myopathy in disease EC |
| 297646017 | 60738003 | Symptomatic inflammatory myopathy in disease NOS |
| 6596981000006117 | 398049005 | MCTD - Mixed connective tissue disease |
| 1591381000006114 | 398049005 | Mixed connective tissue disease |
| 1539141000006113 | 1539141000006109 | Mixed connective tissue disease |

# Supplementary table S2. Age and sex standardised incidence rates (ASIRs) in CPRD using restricted definitions by year, 2012-2023, with 95% confidence intervals (95% CI)

|  | SLE | | SjD | | SSc | | IIM | | MCTD | |
| --- | --- | --- | --- | --- | --- | --- | --- | --- | --- | --- |
| Year | **Incident diagnoses (N)** | **ASIR per 100,000 py (95% CI)** | **Incident diagnoses (N)** | **ASIR per 100,000 py (95% CI)** | **Incident diagnoses (N)** | **ASIR per 100,000 py (95% CI)** | **Incident diagnoses (N)** | **ASIR per 100,000 py (95% CI)** | **Incident diagnoses (N)** | **ASIR per 100,000 py (95% CI)** |
| 2012 | 391 | 3.80 (3.42 - 4.18) | 592 | 5.92 (5.44 - 6.40) | 178 | 1.79 (1.52 - 2.05) | 212 | 2.16 (1.87 - 2.46) | 66 | 0.64 (0.49 - 0.80) |
| 2013 | 406 | 4.00 (3.61 - 4.39) | 589 | 5.84 (5.36 - 6.31) | 198 | 2.00 (1.72 - 2.28) | 209 | 2.10 (1.81 - 2.38) | 50 | 0.50 (0.36 - 0.64) |
| 2014 | 437 | 4.30 (3.89 - 4.71) | 636 | 6.23 (5.75 - 6.72) | 191 | 1.88 (1.61 - 2.14) | 203 | 2.01 (1.73 - 2.29) | 73 | 0.71 (0.54 - 0.87) |
| 2015 | 383 | 3.74 (3.36 - 4.12) | 702 | 6.72 (6.22 - 7.22) | 208 | 2.01 (1.74 - 2.29) | 236 | 2.29 (1.99 - 2.58) | 62 | 0.60 (0.45 - 0.76) |
| 2016 | 376 | 3.60 (3.23 - 3.97) | 725 | 6.85 (6.35 - 7.35) | 250 | 2.35 (2.06 - 2.64) | 238 | 2.28 (1.99 - 2.57) | 76 | 0.72 (0.56 - 0.89) |
| 2017 | 346 | 3.26 (2.92 - 3.61) | 700 | 6.45 (5.97 - 6.93) | 217 | 2.02 (1.75 - 2.30) | 240 | 2.25 (1.96 - 2.54) | 73 | 0.67 (0.51 - 0.82) |
| 2018 | 425 | 3.95 (3.57 - 4.33) | 733 | 6.65 (6.16 - 7.13) | 213 | 1.96 (1.69 - 2.22) | 249 | 2.30 (2.01 - 2.58) | 72 | 0.66 (0.51 - 0.82) |
| 2019 | 338 | 3.09 (2.76 - 3.42) | 706 | 6.30 (5.83 - 6.77) | 189 | 1.68 (1.44 - 1.92) | 286 | 2.57 (2.27 - 2.87) | 68 | 0.61 (0.46 - 0.75) |
| 2020 | 310 | 2.76 (2.45 - 3.07) | 394 | 3.42 (3.08 - 3.76) | 105 | 0.91 (0.73 - 1.08) | 261 | 2.29 (2.02 - 2.57) | 69 | 0.61 (0.47 - 0.76) |
| 2021 | 341 | 3.06 (2.73 - 3.38) | 464 | 4.00 (3.63 - 4.36) | 139 | 1.20 (1.00 - 1.40) | 303 | 2.64 (2.34 - 2.94) | 77 | 0.69 (0.54 - 0.85) |
| 2022 | 302 | 2.63 (2.33 - 2.92) | 465 | 3.90 (3.55 - 4.26) | 152 | 1.29 (1.08 - 1.49) | 278 | 2.37 (2.09 - 2.65) | 71 | 0.59 (0.46 - 0.73) |
| 2023 | 323 | 2.83 (2.52 - 3.14) | 480 | 4.03 (3.67 - 4.39) | 144 | 1.20 (1.01 - 1.40) | 288 | 2.43 (2.15 - 2.71) | 72 | 0.61 (0.47 - 0.75) |

# Supplementary table S3. Number of observed deaths, expected deaths and standardised mortality ratios over the study period by CTD, ag group and sex. Counts less than eight were redacted (*).

|  | SLE | | | SjD | | | SSc | | | IIM | | | MCTD | | |
| --- | --- | --- | --- | --- | --- | --- | --- | --- | --- | --- | --- | --- | --- | --- | --- |
| Age group | Observed deaths (N) | Expected deaths (N) | **SMR (95% CI)** | Observed deaths (N) | Expected deaths (N) | **SMR (95% CI)** | Observed deaths (N) | Expected deaths (N) | **SMR (95% CI)** | Observed deaths (N) | Expected deaths (N) | **SMR (95% CI)** | Observed deaths (N) | Expected deaths (N) | **SMR (95% CI)** |
| Overall |  |  |  |  |  |  |  |  |  |  |  |  |  |  |  |
| 18-39 | 13 | 4 | 3.23 (1.90 - 5.22) | * | * | 1.52 (0.62 - 3.33) | * | * | 3.81 (1.55 - 8.36) | 15 | 2 | 7.21 (4.39 - 11.29) | * | * | 6.21 (2.52 - 13.62) |
| 40-69 | 138 | 77 | 1.79 (1.52 - 2.11) | * | * | 0.96 (0.83 - 1.11) | * | * | 3.20 (2.76 - 3.69) | 258 | 76 | 3.39 (3.01 - 3.82) | * | * | 1.63 (1.10 - 2.34) |
| 70+ | 234 | 216 | 1.08 (0.95 - 1.22) | * | * | 0.64 (0.59 - 0.70) | * | * | 1.41 (1.27 - 1.56) | 482 | 406 | 1.19 (1.09 - 1.29) | * | * | 1.33 (0.97 - 1.78) |
| Total | 385 | 297 | 1.30 (1.17 - 1.43) | 730 | 1038 | 0.70 (0.65 - 0.76) | 531 | 305 | 1.74 (1.60 - 1.89) | 755 | 484 | 1.56 (1.45 - 1.67) | 67 | 45 | 1.50 (1.18 - 1.88) |
| Males |  |  |  |  |  |  |  |  |  |  |  |  |  |  |  |
| 18-39 | * | * | 1.92 (0.46 - 7.07) | * | * | * | * | * | 3.31 (0.80 - 12.21) | * | * | 6.84 (3.64 - 11.98) | * | * | * |
| 40-69 | * | * | 2.02 (1.43 - 2.79) | * | * | 1.08 (0.76 - 1.51) | * | * | 2.81 (2.07 - 3.75) | * | * | 2.82 (2.36 - 3.34) | * | * | 1.14 (0.46 - 2.50) |
| 70+ | * | * | 1.29 (1.01 - 1.63) | * | * | 0.75 (0.61 - 0.90) | * | * | 1.55 (1.23 - 1.93) | * | * | 1.24 (1.10 - 1.40) | * | * | 2.17 (1.25 - 3.56) |
| Total | 94 | 64 | 1.47 (1.20 - 1.79) | 128 | 159 | 0.80 (0.68 - 0.95) | 113 | 61 | 1.85 (1.54 - 2.21) | 395 | 256 | 1.54 (1.40 - 1.70) | 16 | 9 | 1.77 (1.09 - 2.73) |
| Females |  |  |  |  |  |  |  |  |  |  |  |  |  |  |  |
| 18-39 | * | * | 3.43 (1.98 - 5.63) | * | * | 1.73 (0.70 - 3.79) | * | * | 4.02 (1.46 - 9.67) | * | * | 7.84 (3.68 - 15.26) | * | * | 7.67 (3.11 - 16.81) |
| 40-69 | * | * | 1.74 (1.44 - 2.08) | * | * | 0.94 (0.80 - 1.10) | * | * | 3.34 (2.82 - 3.93) | * | * | 4.17 (3.52 - 4.90) | * | * | 1.78 (1.16 - 2.64) |
| 70+ | * | * | 1.02 (0.88 - 1.18) | * | * | 0.63 (0.57 - 0.68) | * | * | 1.38 (1.23 - 1.55) | * | * | 1.12 (0.98 - 1.28) | * | * | 1.14 (0.78 - 1.60) |
| Total | 291 | 233 | 1.25 (1.11 - 1.39) | 602 | 878 | 0.69 (0.63 - 0.74) | 418 | 244 | 1.71 (1.56 - 1.88) | 360 | 228 | 1.58 (1.42 - 1.75) | 51 | 36 | 1.43 (1.09 - 1.85) |

# Supplementary table S4. Age and sex standardised mortality rates for incident CTD diagnoses excluding cases with more than one CTD diagnosis (before or during our study period).

|  | SLE | SjD | SSc | IIM | MCTD |
| --- | --- | --- | --- | --- | --- |
| Number of deaths | 331 | 665 | 471 | 713 | 46 |
| Total person-years | 21523 | 45135.65 | 12480.42 | 17312.16 | 3018.456 |
| ASMR per 1,000 person-years | 16.77 (14.93 - 18.61) | 9.61 (8.58 - 10.64) | 24.42 (21.97 - 26.87) | 27.85 (25.57 - 30.14) | 16.26 (11.41 - 21.11) |

# Supplementary table S5. Standardised mortality ratios (SMRs) excluding cases with more than one CTD diagnosis. Displayed by CTD and sex.

|  | **SLE** | | | **SjD** | | | **SSc** | | | **IIM** | | | **MCTD** | | |
| --- | --- | --- | --- | --- | --- | --- | --- | --- | --- | --- | --- | --- | --- | --- | --- |
| Age group | Observed deaths (N) | Expected deaths (N) | **SMR (95% CI)** | Observed deaths (N) | Expected deaths (N) | **SMR (95% CI)** | Observed deaths (N) | Expected deaths (N) | **SMR (95% CI)** | Observed deaths (N) | Expected deaths (N) | **SMR (95% CI)** | Observed deaths (N) | Expected deaths (N) | **SMR (95% CI)** |
| **Overall** | 331 | 261 | 1.27 (1.14 - 1.41) | 666 | 980 | 0.68 (0.63 - 0.73) | 471 | 275 | 1.71 (1.56 - 1.87) | 713 | 467 | 1.53 (1.42 - 1.64) | 46 | 33 | 1.40 (1.05 - 1.83) |
| **Males** | 84 | 58 | 1.44 (1.16 - 1.76) | 123 | 156 | 0.79 (0.66 - 0.93) | 104 | 58 | 1.80 (1.49 - 2.16) | 386 | 253 | 1.53 (1.38 - 1.68) | 11 | 7 | 1.48 (0.83 - 2.47) |
| **Females** | 247 | 203 | 1.22 (1.08 - 1.38) | 543 | 823 | 0.66 (0.61 - 0.72) | 367 | 217 | 1.69 (1.52 - 1.86) | 327 | 214 | 1.53 (1.37 - 1.70) | 35 | 25 | 1.38 (0.99 - 1.87) |

# Supplementary table S6. Age and sex standardised mortality rates for incident CTD diagnoses from 2012 to 2019 (pre-COVID-19 pandemic).

|  | SLE | SjD | SSc | IIM | MCTD |
| --- | --- | --- | --- | --- | --- |
| Number of deaths | 174 | 308 | 261 | 359 | 24 |
| Total person-years | 12271.8 | 23263.4 | 7049.6 | 8419.9 | 1985.9 |
| ASMR per 1,000 person-years | 16.07 (13.61 - 18.53) | 9.47 (8.08 - 10.87) | 25.89 (22.40 - 29.39) | 29.56 (26.29 - 32.83) | 15.11 (8.07 - 22.15) |

# Supplementary table S7. Age and sex standardised mortality rates for incident CTD diagnoses from 2020 to 2023 (post-COVID-19 pandemic).

|  | SLE | SjD | SSc | IIM | MCTD |
| --- | --- | --- | --- | --- | --- |
| Number of deaths | 44 | 44 | 61 | 156 | 14 |
| Total person-years | 2620.9 | 4643.8 | 1226.6 | 2954.6 | 597.9 |
| ASMR per 1,000 person-years | 21.35 (14.88 - 27.83) | 8.28 (4.89 - 11.67) | 34.45 (25.56 - 43.34) | 38.71 (32.10 - 45.31) | 26.45 (11.27 - 41.63) |

# Supplementary table S8. Standardised mortality ratios (SMRs) from 2012 – 2019 (pre-COVID-19 pandemic). Displayed by CTD and sex. Counts less than eight were redacted (*).

|  | **SLE** | | | **SjD** | | | **SSc** | | | **IIM** | | | **MCTD** | | |
| --- | --- | --- | --- | --- | --- | --- | --- | --- | --- | --- | --- | --- | --- | --- | --- |
| Age group | Observed deaths (N) | Expected deaths (N) | **SMR (95% CI)** | Observed deaths (N) | Expected deaths (N) | **SMR (95% CI)** | Observed deaths (N) | Expected deaths (N) | **SMR (95% CI)** | Observed deaths (N) | Expected deaths (N) | **SMR (95% CI)** | Observed deaths (N) | Expected deaths (N) | **SMR (95% CI)** |
| **Overall** | 174 | 128 | 1.35 (1.17 - 1.56) | 308 | 431 | 0.72 (0.64 - 0.80) | 261 | 133 | 1.97 (1.74 - 2.22) | 359 | 197 | 1.82 (1.64 - 2.02) | 24 | 17 | 1.43 (0.96 - 2.06) |
| **Males** | 43 | 28 | 1.55 (1.15 - 2.04) | 57 | 67 | 0.85 (0.65 - 1.08) | 59 | 27 | 2.21 (1.71 - 2.80) | 187 | 107 | 1.75 (1.52 - 2.01) | * | * | 1.75 (0.82 - 3.41) |
| **Females** | 131 | 101 | 1.30 (1.10 - 1.53) | 251 | 363 | 0.69 (0.61 - 0.78) | 202 | 106 | 1.91 (1.66 - 2.18) | 172 | 90 | 1.91 (1.65 - 2.21) | * | * | 1.35 (0.86 - 2.04) |

# Supplementary table S9. Standardised mortality ratios (SMRs) from 2020 - 2023 (post-COVID-19 pandemic). Displayed by CTD and sex. Counts less than eight were redacted (*).

|  | **SLE** | | | **SjD** | | | **SSc** | | | **IIM** | | | **MCTD** | | |
| --- | --- | --- | --- | --- | --- | --- | --- | --- | --- | --- | --- | --- | --- | --- | --- |
| Age group | Observed deaths (N) | Expected deaths (N) | **SMR (95% CI)** | Observed deaths (N) | Expected deaths (N) | **SMR (95% CI)** | Observed deaths (N) | Expected deaths (N) | **SMR (95% CI)** | Observed deaths (N) | Expected deaths (N) | **SMR (95% CI)** | Observed deaths (N) | Expected deaths (N) | **SMR (95% CI)** |
| **Overall** | 44 | 28 | 1.58 (1.18 - 2.08) | 44 | 77 | 0.57 (0.43 - 0.76) | 61 | 27 | 2.28 (1.78 - 2.89) | 156 | 68 | 2.29 (1.95 - 2.66) | 14 | 6 | 2.39 (1.43 - 3.79) |
| **Males** | 16 | 7 | 2.46 (1.52 - 3.80) | 10 | 12 | 0.82 (0.45 - 1.40) | 14 | 5 | 2.70 (1.62 - 4.29) | 90 | 35 | 2.55 (2.07 - 3.10) | * | * | 2.01 (0.73 - 4.84) |
| **Females** | 28 | 21 | 1.31 (0.91 - 1.84) | 34 | 64 | 0.53 (0.38 - 0.72) | 47 | 22 | 2.18 (1.64 - 2.85) | 66 | 33 | 2.00 (1.58 - 2.52) | * | * | 2.51 (1.42 - 4.20) |

# Supplementary figure S1. Individual graphs of age standardised incidence rates (ASIRs) by sex, with 95% confidence intervals (95% CI). 1a. SLE, 1b. SjD, 1c. SSc, 1d. IIM, 1e. MCTD.


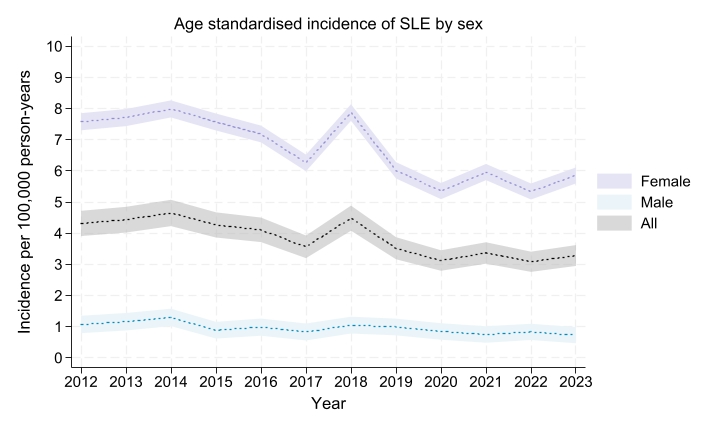


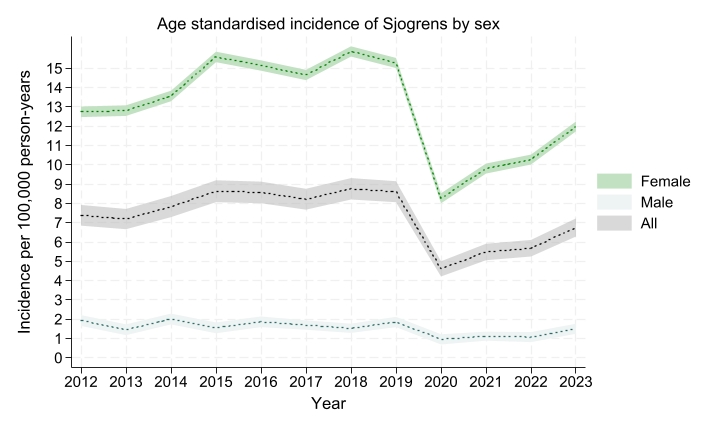


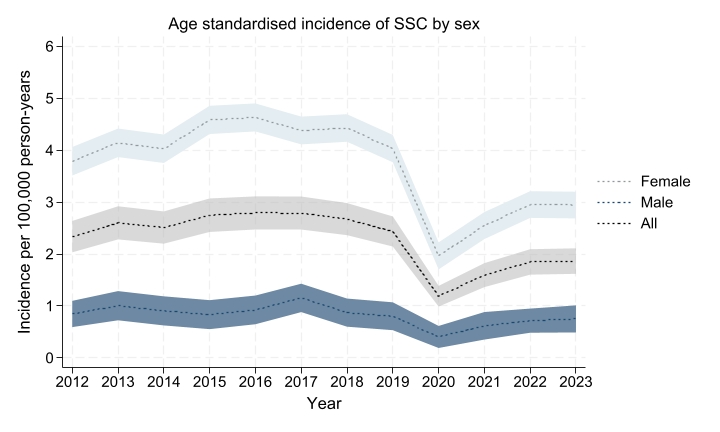


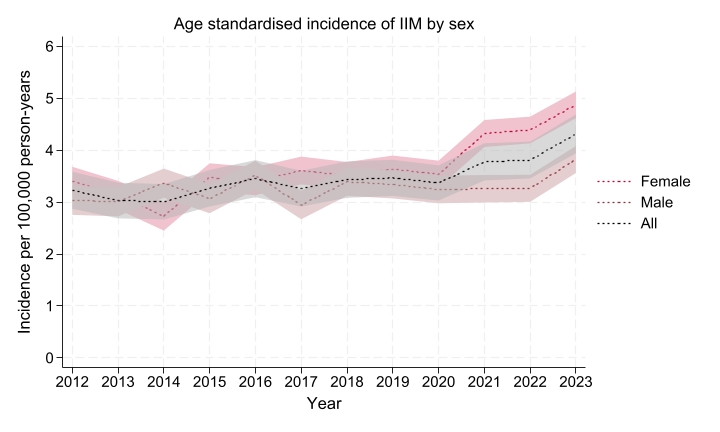


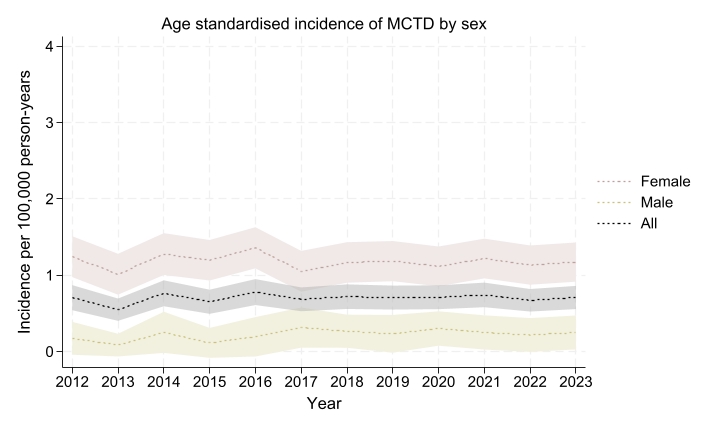


# Supplementary figure S2. Age and sex standardised incidence rates (ASIRs) using restricted definitions in CPRD by year, 2012-2023, with 95% confidence intervals (95% CI).
